# Relationship Between Serum Hydrogen Sulfide and Ferritin Levels in Type 2 Diabetes Mellitus: A Retrospective Study at a Tertiary Hospital in Eastern India

**DOI:** 10.7759/cureus.82815

**Published:** 2025-04-22

**Authors:** Pinaki Saha, Bikramaditya Mukherjee, Sayantan Dasgupta, Santanu Sen, Utpal Biswas

**Affiliations:** 1 Biochemistry, Kali Pradip Chaudhuri (KPC) Medical College, Kolkata, IND; 2 Biochemistry, Manipal Tata Medical College, Manipal Academy of Higher Education, Jamshedpur, IND; 3 Biochemistry, North Bengal Medical College and Hospital, Siliguri, IND; 4 Biochemistry, Calcutta National Medical College and Hospital, Kolkata, IND

**Keywords:** diabetes mellitus, ferritin, hydrogen sulfide, oxidative stress, type 2 diabetes

## Abstract

Introduction: Diabetes mellitus, one of the widespread endocrine disorders, is characterized by hyperglycemia. Hydrogen sulfide (H_2_S), a signaling gas transmitter, is involved in carbohydrate metabolism. The pathophysiology of β-cell dysfunction in type 1 and 2 diabetes may be significantly affected by alterations in the H_2_S balance. Diabetes mellitus causes an increase in ferritin, an acute-phase reactant.

Aims and objectives: This study aims to investigate the relationship between serum H_2_S and ferritin levels in patients with type 2 diabetes.

Methodology: Spectrophotometric measurements of serum H_2_S were made using a lab-standardized technique. Enzyme-linked immunosorbent assay (ELISA) was used with standardized kits (BioRad Laboratories, Hercules, CA, USA) to determine serum ferritin levels.

Results: Compared to the control group, type 2 diabetic patients had significantly greater serum ferritin levels and significantly lower H_2_S levels. H_2_S and ferritin levels are negatively correlated among cases and controls.

Conclusion: The negative connection between ferritin and H_2_S levels indicates that oxidative stress plays an important part in the beginning and development of diabetes mellitus.

## Introduction

Diabetes mellitus is a spectrum of ailments in which there is hyperglycemia, and its prevalence has increased dramatically in the last 20 years [1-3]. Hydrogen sulfide (H_2_S) is endogenously produced from the amino acid L-cysteine by the enzymes cystathionine beta synthase (CBS), cystathionine gamma lyase (CSE), and 3-mercaptopyruvate sulfur transferase in mammalian tissues. Alterations in the levels of these enzymes can have a marked effect on the pathophysiology of β-cell dysfunction in both type 1 and type 2 diabetes [4]. Alterations in H_2_S homeostasis also influence the endothelial damage caused by persistently or intermittently high blood glucose levels in diabetes [2]. It has been suggested by other studies that H_2_S deficiency leads to diabetic nephropathy and cardiomyopathy through oxidative stress modulation [5].

Ferritin is a 24-subunit protein found throughout the body and is the soluble, nontoxic form of iron, which is the total iron storage in the body. Nonspecifically, ferritin levels rise, and it is recognized as an indicator of both acute and chronic inflammation and acts as an acute phase reactant [6-10]. Our previous study showed a positive correlation between serum ferritin levels and total oxidative stress (TOS) and a negative correlation with total antioxidant defense (TAD) levels [11].

The clinical importance of understanding this inverse relationship between ferritin and H_2_S biomarkers becomes significant because of their fundamental roles in type 2 diabetes mellitus (T2DM) pathophysiology. Research shows ferritin functions as an iron-storage protein, yet medical experts now identify it as a pro-inflammatory marker that contributes to insulin resistance and diabetic complication development [12]. H_2_S protects endothelial function as well as oxidative stress and protects from damage to mitochondria, which contributes to the development of diabetic nephropathy, cardiomyopathy, and neuropathy [13]. The negative correlation between these measurements suggests widespread inflammation and redox imbalances occur in diabetic patients. The combination of ferritin and H_2_S measurements shows potential for better predicting vascular and microvascular complications, according to diagnostic research [14]. Stratification of patients for preventive interventions would become more effective because of this ability. Endogenous H_2_S levels could be pharmacologically or enzymatically increased via supplements and regulators as a new supportive treatment method to fight diabetes-induced inflammation and oxidative damage [15]. The early discovery of H_2_S imbalances in T2DM patients combined with proper treatment adjustments can potentially reduce disease severity and control complication development to improve both short-term outcomes and long-term life quality for people managing T2DM [16]. The experimental findings establish an essential biochemical link that provides a basis for future medical research in individualized diabetes management.

Objectives

The goal was to determine whether ferritin and serum H_2_S levels in patients with type 2 diabetes were correlated in any way, with the broader aim of assessing whether this relationship could serve as a potential biomarker profile for early detection of metabolic dysregulation, stratification of inflammatory burden, or identification of patients at higher risk for complications. Establishing such a correlation could inform the development of novel diagnostic tools or therapeutic strategies that target oxidative stress and inflammation in diabetes management.

## Materials and methods

The study enrolled 40 patients who had T2DM between 20 and 50 years old alongside 40 control participants who matched the diabetic patients regarding age and sex yet had normal fasting blood glucose levels. The study recruited participants through convenience sampling at Nilratan Sircar (NRS) Medical College, Kolkata's outpatient and inpatient departments, throughout six months of 2015. A pilot investigation determined the sample size through practical constraints and previous exploratory studies about biochemical changes in metabolic conditions. The research sought to establish a connection between H_2_S levels in serum and ferritin measurements in diabetic patients while examining TOS and TAD markers for diagnostic or prognostic value assessment.

The research excluded patients who had medical conditions that could independently affect oxidative stress or inflammatory markers. Research participants were excluded if they had polycystic ovarian syndrome, malignant diseases, chronic renal failure, pregnancy, other endocrine disorders, or were taking antioxidant supplements or medications affecting endogenous H_2_S metabolism. The study excluded these conditions and interventions because they independently modify oxidative stress and inflammatory pathways that could distort the analysis of serum ferritin and H_2_S levels. The hormonal changes in PCOS and pregnancy affect metabolic and redox homeostasis, while malignancies and chronic renal failure lead to chronic inflammation and iron metabolism disturbances, and antioxidants or H_2_S-altering agents could affect the biochemical markers, which weakens the internal validity of the correlation analysis. Moreover, the study received approval from the Institutional Ethics Committee of NRS Medical College through their decision NMC/490 during their 24-01-2013 meeting. Each participant signed written informed consent before joining the study. The research followed institutional ethical guidelines precisely while reporting study progress updates to the ethics committee according to their requirements.

Ferritin, TOS, and TAD levels in patients with type 2 diabetes were further investigated as a pilot project in this research. The study excluded patients with conditions such as polycystic ovarian syndrome, malignant diseases, renal failure, pregnant women, other endocrine disorders, and patients taking antioxidant or H_2_S-modifying medications.

Collection of samples

Five ml of whole blood was drawn aseptically from a superficial vein following an overnight fast of 8 to 10 hours. Three ml was taken in a plain vial with a clot activator, and 2 ml was taken in a fluoride vial and centrifuged for five minutes at 2500 rpm. Different biochemical parameters were estimated using separated serum, while fasting plasma glucose was estimated using plasma.

Measurement of H_2_S concentration in serum

Methods previously described were modified and standardized in our laboratory to assess serum H_2_S [4,12]. In the presence of the oxidizing agent Fe^3+^ in hydrochloric acid, sulfide combines with N, N-dimethyl-p-phenylenediamine sulfate to create methylene blue, whose absorbance is measured at 670 nm using an ultraviolet (UV)-Vis dual-beam spectrophotometer (Agilent Technologies, Santa Clara, CA, USA).

Assay procedure

In a sealed glass tube, 425 µl of phosphate-buffered saline (PBS), 250 µl of 10% trichloroacetic acid, and 75 µl of serum were combined. After that, the mixture was centrifuged for 30 minutes at 3000 rpm. After that, the resulting solution was gathered in a separate tube and combined with 60 µl of 10% sodium hydroxide (NaOH), 250 µl of 1% zinc acetate, 133 µl of 20 millimolar N, N-dimethyl-p-phenylenediamine sulfate in 7.2 mM HCl, and 133 µl of 30 millimolar ferric chloride (FeCl_3_) in 1.2 mM HCl. After being sealed, this tube was allowed to sit at room temperature for 10 minutes. Every sample was analyzed three times, and a calibration curve made using sodium sulfide (NaHS, Sigma-Aldrich, MO, USA) was used to determine the serum H_2_S levels ranging from 25 to 250 µmol/l. The maximum sensitivity of this technique was 25 µmol/l, and its intra- and inter-assay variances were 7.576 and 3.944, respectively [13].

Assay of serum ferritin levels and additional parameters

Conventional enzyme-linked immunosorbent assay (ELISA) kits (BioRad Laboratories, Hercules, CA, USA) were used to assess serum ferritin. The glucose oxidase-peroxidase method was used to measure blood glucose levels utilizing a fully automated biochemistry analyzer and a standardized kit following two stages of external quality control.

Statistical analysis

Because the parameters were regularly distributed, the data were displayed as mean ± standard deviation (SD). IBM SPSS Statistics for Windows, Version 20 (Released 2011; IBM Corp., Armonk, New York, United States) was used a t-test for independence and Pearson's correlation to compare the data. The analysis considered a p-value of 0.05 or less to be significant.

## Results

Table 1 summarizes the demographic and biochemical characteristics of the study participants.

**Table 1:** Demographic and biochemical characteristics of the study Hyphens (-) in the table represent missing or inapplicable data. For instance, some variables may not be relevant for certain groups (e.g., age-related conditions or data not collected for specific reasons). H_2_S: hydrogen sulfide

Variables	Cases (Mean ± SD)	Controls (Mean ± SD)	p-value
Age (years)	42.9 ± 5.6	43.3 ± 6.1	-
Sex (M/F)	22/18	24/16	-
Body mass index (BMI)	24.0 ± 3.9	24.9 ± 2.5	-
Fasting blood glucose (mg/dl)	179.3 ± 27.8	79.1 ± 12.3	<0.05
Serum ferritin (μg/l)	168.7 ± 32.7	21.5 ± 8.6	<0.001
Serum H_2_S (μmol/l)	32.9 ± 4.03	62.9 ± 10.9	<0.05

**Figure 1:**
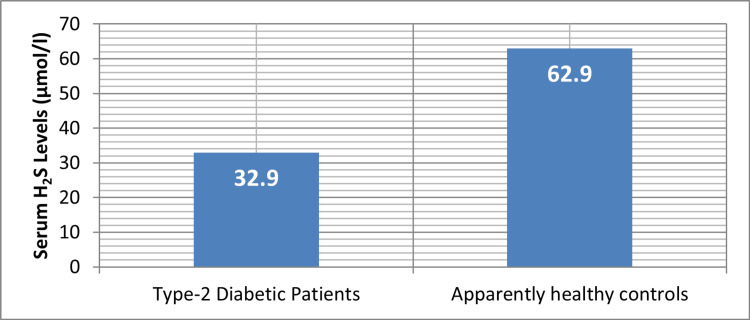
Mean values of H2S levels in serum of recently diagnosed patients suffering from type 2 diabetes mellitus and healthy controls Image credit: Bikramaditya Mukherjee This figure shows that type 2 diabetes mellitus patients who received recent diagnoses have lower serum H_2_S levels (mean = 32.9 μmol/l) than healthy controls (mean = 62.9 μmol/l). The substantial decrease in H_2_S levels indicates there might be a relationship between this change and type 2 diabetes pathophysiology. H_2_S: hydrogen sulfide

**Figure 2:**
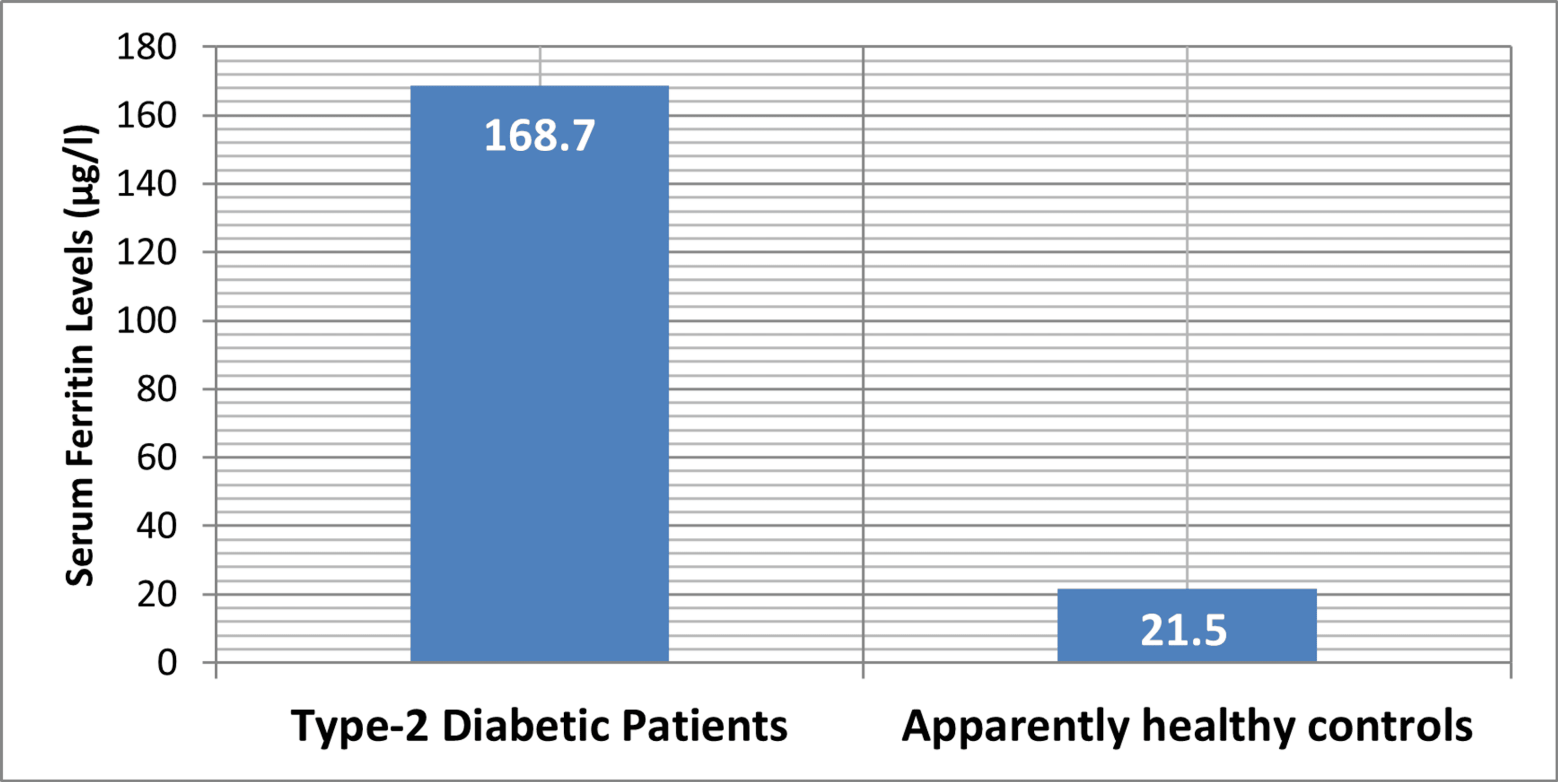
Ferritin levels in the serum of newly diagnosed type 2 diabetes mellitus patients and seemingly healthy controls on average Image credit: Bikramaditya Mukherjee This figure shows that patients with type 2 diabetes mellitus have significantly higher serum ferritin levels (mean = 168.7 µg/l) than apparently healthy controls (mean = 21.5 µg/l). Ferritin levels rise substantially in diabetic patients because systemic inflammation and oxidative stress contribute to type 2 diabetes pathogenesis.

**Figure 3:**
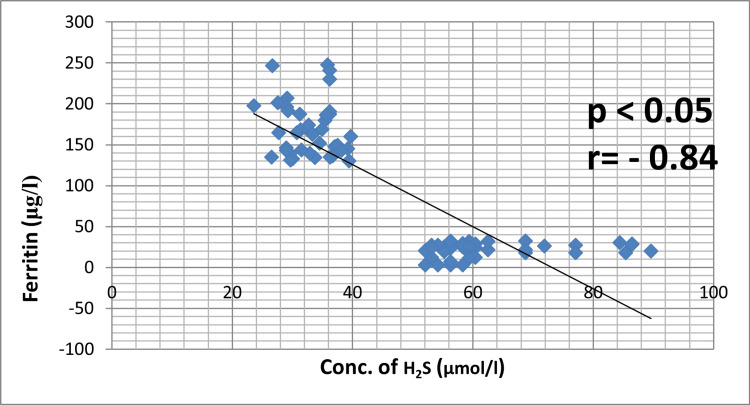
An illustration showing the relationship between the serum ferritin and H2S levels of newly identified type 2 diabetes mellitus patients and seemingly healthy controls Image credit: Bikramaditya Mukherjee H_2_S: hydrogen sulfide

According to our research, patients with a new diagnosis of T2DM had a substantially higher mean fasting plasma glucose level (179.3 ± 27.8 mg/dl; p < 0.05) than seemingly healthy subjects (79.1 ± 12.3 mg/dl) (see Table 1). Figure 1 shows the mean values of H_2_S levels in serum for patients with T2DM and healthy controls. The patients' mean serum H_2_S levels were much lower than those of the controls (62.9 ± 10.9 μmol/l; p < 0.05) at 32.9 ± 4.03 μmol/l. Furthermore, compared to the control group (21.5 ± 8.6 μg/l), the total serum ferritin levels of these individuals were significantly higher (168.7 ± 32.7 μg/l; p < 0.001) (Figure 2). Figure 3 depicts the relationship between serum ferritin and H_2_S levels in patients with T2DM and healthy controls.

A substantial negative correlation (r = -0.84, p < 0.05) has been found in our study between H_2_S and ferritin levels.

## Discussion

Serum ferritin levels were significantly higher in newly diagnosed type 2 diabetes patients than in healthy controls. These findings are in line with earlier studies. Oxidative stress, which is caused by hyperferritinemia, damages pancreatic β-cells. This damage prevents the liver from getting insulin and from insulin effectively reducing hepatic glucose production [14]. In red blood cells, hyperglycemia leads to an irreversible, non-enzymatic contact between glucose and hemoglobin, and the glycation process releases free iron [15]. This process often triggers reduction-oxidation processes that generate free radicals such as reactive oxygen species (ROS). These radicals can injure pancreatic β-cells, exacerbate the inflammatory response, oxidatively damage macromolecules, and cause serum ferritin levels to rise [16,17].

The results of this study showed that people with T2DM had much lower amounts of H_2_S in their blood compared to healthy people. Hyperglycemia and oxidative stress were suggested to be involved in this deficiency by some research. The reduced H_2_S levels may be due to increased H_2_S consumption by hyperglycemic cells, which is caused by ROS produced by the mitochondria [18]. Another underlying cause could be the downregulation or inactivation of enzymes that are responsible for the synthesis of H_2_S [19]. Diabetes is related to increased ROS-linked H_2_S consumption and CSE-mediated H_2_S reduction when combined. Nevertheless, the exact mechanism is still unknown and needs to be further investigated for clarification. Serum ferritin levels in T2DM could be used as a predictor of their oxidative state. Our study supported these conclusions, given the negative relationship between ferritin and H_2_S levels. Excessive generation of ROS and progression of oxidative stress occur as a result of chronic hyperglycemia [20-22]. The H_2_S deficit in T2DM patients compared to healthy individuals [23] may lead to oxidative stress and complications of diabetes.

This study indicated that oxidative stress is an important pathogenic factor in T2DM, according to the inverse correlation between H_2_S and ferritin levels. In T2DM patients, the elevated ferritin levels, as a part of the inflammatory response, caused oxidative insult, β-cell dysfunction, and insulin resistance [1,16]. In addition, the rise in ROS due to hyperglycemia aggravated this oxidative stress, and ferritin levels were a good indicator of the disease progression. These findings of an increase in oxidative stress indicated a need to manipulate this process for the effective management of T2DM.

The deleterious effects of oxidative stress were also shown by the reduced levels of H_2_S in T2DM patients. The protective role of the antioxidant gas transmitter H_2_S is to neutralize ROS and prevent cellular damage. Many complications, such as diabetic nephropathy and cardiomyopathy, have been associated with H_2_S deficiency, and it increases oxidative stress in tissues [5,18]. These findings suggest that restoring H_2_S levels may alleviate oxidative damage and improve insulin sensitivity, and thus may be a promising therapeutic target for oxidative stress in T2DM patients.

Study limitations

This was an initial study of a small sample size, and the authors hope to involve more participants and conduct the study with a larger sample size over a longer period.

## Conclusions

The negative correlation between ferritin and H_2_S levels in type 2 diabetes suggests that oxidative stress plays a crucial role in the disease's development. Increased ferritin levels indicate chronic inflammation and oxidative damage, which impair insulin production and action. The reduced H_2_S levels further support the notion of compromised antioxidant defense, worsening the oxidative stress. Together, these findings highlight the importance of oxidative stress in T2D progression and its potential as a therapeutic target.
